# Multiple Viral Infection Detected from Influenza-Like Illness Cases in Indonesia

**DOI:** 10.1155/2017/9541619

**Published:** 2017-01-23

**Authors:** Kindi Adam, Krisna Nur Andriana Pangesti, Vivi Setiawaty

**Affiliations:** Research and Development Center for Biomedical and Basic Health Technology, Jakarta, Indonesia

## Abstract

Influenza is one of the common etiologies of the upper respiratory tract infection (URTI). However, influenza virus only contributes about 20 percent of influenza-like illness patients. The aim of the study is to investigate the other viral etiologies from ILI cases in Indonesia. Of the 334 samples, 266 samples (78%) were positive at least for one virus, including 107 (42%) cases of multiple infections. Influenza virus is the most detected virus. The most frequent combination of viruses identified was adenovirus and human rhinovirus. This recent study demonstrated high detection rate of several respiratory viruses from ILI cases in Indonesia. Further studies to determine the relationship between viruses and clinical features are needed to improve respiratory disease control program.

## 1. Introduction

Lower respiratory tract infection (LRTI) remains one of the major causes of mortality and morbidity in children under five years globally [1]. Viruses have already been recognized as important etiologies of respiratory infections with influenza virus which is considered as the main contributor. The epidemiology and public health impact of influenza infections are relatively well described as many studies and surveillance have been conducted in part of pandemic preparedness [2–5]. Most of the countries in the world, including Indonesia, have developed influenza surveillance, influenza-like illness (ILI) surveillance, and severe acute respiratory illness (SARI), which form the network under WHO through Global Influenza Surveillance and Response Systems (GISRS) [4–8]. This network improves influenza disease control by providing support on influenza vaccine recommendation, laboratory diagnostic tools, antiviral, and public health risk assessment. As influenza virus contributed only less than 30 percent of viral respiratory infections, there is an urge to investigate the contribution of other respiratory viruses for improving respiratory disease control program [6].

Recent advancement of molecular technology supports the investigation and characterization of several respiratory viruses. The molecular technology improves the capability to study respiratory viruses, which are previously identified: rhinovirus, adenovirus, respiratory syncytial viruses, parainfluenza virus, and also the new emerging viruses/strain viruses: MERS coronavirus, human metapneumovirus, and human rhinovirus strain C. Multiple detection platforms, which recently have been developed, allows relatively inexpensive and timely detection of several viruses [9–11]. The detection of multiple respiratory viruses will accommodate the efforts to determine the epidemiology of noninfluenza respiratory viruses in the community, which will further help the respiratory disease control program including the use of antimicrobial agents [12].

Previous results of the investigation on acute respiratory infection patients in several countries showed the difference in the prevalence of respiratory viruses among studies [3, 13–15]. Study design including the case definition, study population, time of the study, and diagnostic tools being used have been considered as factors that influenced the variation [16]. Each virus has different seasonality circulation and an age-related prevalence that can lead to a specific pattern of virus cocirculation in many studies [17–19]. Moreover, the occurrence of virus coinfection in which two or more viruses are detected in a single patient has been described in recent studies using multiple pathogen detection platforms [20–22].

There are limited studies on viral pathogens of respiratory tract infection in low-middle income countries including Indonesia. Previous studies have been conducted mostly focused on specific viral pathogens, especially influenza [6, 23, 24]. The prevalence of noninfluenza respiratory infections is relatively unknown. Therefore, this study has an objective to investigate the prevalence of viral etiologies from ILI cases in Indonesia.

## 2. Method

### 2.1. Study Design and Samples

**Figure 1:**
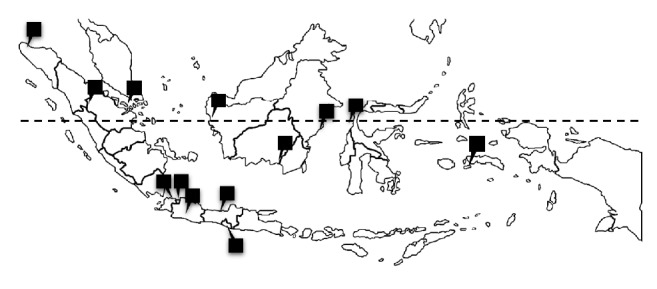
Location of ILI sentinels in 13 provinces in Indonesia.

Influenza-like illness (ILI) surveillance was conducted in thirteen sentinels' public health center across 13 Indonesia provinces in 2012 in Figure 1. The WHO case definitions were used to determine the ILI cases: fever ≥ 38°C and a cough or a sore throat [25]. Demographic and clinical data were obtained from questionnaires that are filled out by trained staff. Throat swab and nasal swabs from 1,692 patients were collected in viral transport medium (VTM), which were then sent to the Virology Laboratory, National Institute of Health Research and Development (NIHRD) in Jakarta. The VTM consists of bovine serum albumin, penicillin, streptomycin, and amphotericin B, according to WHO surveillance manual [6]. Only specimens were received by the laboratory within three days after the collection was processed for molecular examinations. Specimens were stored in a −80 freezer prior to the laboratory tests. For this multiplex study, a total of three hundred thirty-four specimens were randomly selected.

### 2.2. Nucleic Acid Extraction and Multiplex PCR

Total viral nucleic acid was extracted from 190 *μ*l of viral transport medium using RiboSpin v_RD GeneAll extraction Kit from Seegene (Seegene Inc., Seoul, South Korea). A 10 *μ*l internal control which is inserted in the package was added to each of the 190 *μ*l samples for internal amplification control to check the PCR process. 40 *μ*l elution buffer was added according to the manufacturer instructions. Seeplex® RV16 ACE Multiplex detection (Seegene Inc., Seoul, South Korea), a multiplex real time PCR platform, which is able to detect 16 viruses including human adenovirus (ADV), influenza A and B viruses (Flu A, Flu B), human parainfluenza viruses 1/2/3/4 (PIV 1/2/3/4), human rhinoviruses A/B/C (RV A/B/C), human respiratory syncytial viruses A and B (RSV A, RSV B), human bocaviruses 1/2/3/4 (BoV1/2/3/4), human coronaviruses 229E, NL63 and OC43 (CoV-229E, CoV-NL63, and CoV-OC43), human metapneumovirus (hMPV), and human enterovirus (EV) was used. The protocol followed manufacturer's instruction as described before [26].

cDNA synthesis process was performed using cDNA synthesis premix (Seegene Inc., Seoul, South Korea) with 8 *μ*l of RNA, 2 *μ*l random hexamer primer, and 10 *μ*l mix of transcriptase, MgCl_2_, dNTP, and buffer. The multiplex reaction was performed using Biorad CFX 96 Real Time Thermal Cycler. The reaction mixture was first denatured at 95°C for 15 min, followed by 50 cycles of denaturation at 95°C for 30 s, annealing at 60°C for 60 s, extension at 72°C for 30 s, and a final extension step at 55°C for 30 s. The melting curve temperature from 55°C to 85°C (5 s/0, 5°C) was used to read the amplification. Any positive result was detected as a peak in electropherogram, compared to positive control.

### 2.3. Statistical Analysis

The descriptive statistics were used to analyse demographics, clinical, and laboratory data using Microsoft Excel (Microsoft Corporation, Washington, US). To compare between single and multiple infection, two paired tests were used.

### 2.4. Ethical Approval

This study has been approved by the Health Research Ethics Committee, National Institute of Health Research and Development, Ministry of Health, Indonesia.

## 3. Results and Discussion

This present study is the first study reporting respiratory viruses' detection in ILI patients in Indonesia. During 2012, 1692 patients that meet ILI case definition criteria were enrolled. From 334 cases randomly selected, 175 (52.3%) were male and 159 (47.6%) were female. The median age was nine years with a range from 1 month to 79 years. Most of the cases selected were patients with age > 5 years old (60.7%).

Two hundred and fifty-six (76.6%) specimens contained one or more viruses. Single infection was detected in 138 (41.32%) cases and multiple infections were identified in 105 (31.4%) samples. The high detection rate of respiratory viruses from ILI cases in this study is similar to the previous studies in other countries including Cameroon (65.06%), Nanjing China (50.6%), and Cambodia (35.5%) [3, 14, 15]. However, this result is higher compared to the previous results which focused on hospitalized patients in Indonesia: 27% and 8.2% [23, 27]. The difference of viral detection rate among outpatient ILI cases and inpatient is most likely due to the time of infection. Hospitalized patients who suffer from lower respiratory tract infection might experience viral infection first which is then followed by bacterial infection later.

Of these positive specimens, influenza virus is the most detected virus (36.1%), followed by human rhinovirus and human adenovirus.  Table 1 shows the distribution of respiratory viruses associated with ILI cases in 2012. The high prevalence of influenza virus detected from ILI cases in this recent study is concordant with several results from other countries [3, 15, 28]. Previous results in hospitalized suspected influenza patients in Indonesia demonstrated similar results as influenza is the most detected virus.

The difference in the prevalence of viruses identified in the ILI specimens among studies has been recognized. Time of samples collection and the composition of age in the study population are assumed to influence the proportion of virus identified [3]. Previous results between studies in temperate countries showed the variation of viruses detected based on the seasons [18, 29]. The seasonality of respiratory viruses in tropical region is relatively undefined clearly except for RSV and influenza seasonality which associated with rainy seasons [30]. Therefore, further investigation is needed to determine the seasonality of other respiratory viruses in the tropic region.

**Figure 2:**
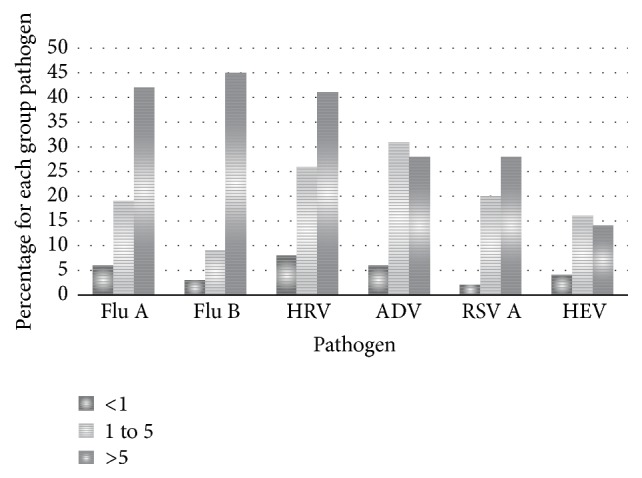
Virus detection by age group from influenza-like illness patients. A total of 27 patients <1 year old, 104 patients aged 1 to 5 years, and 203 patients aged >5 years were tested.

The prevalence of viruses is assumed to be associated with the age of patients. Regarding the age group in this study which is mostly more than 5 years old, the prevalence of RSV was low as RSV is mostly detected in children under one year old. The prevalence of adenovirus and RSV A infections increased with age. This finding is similar to other researches [20]. Influenza was the most prevalent viral infection under-one-year-old group and above-five-year-old group. In the one-to-five-year-old group, adenovirus was the most prevalent.  Figure 2 also shows the number of respiratory virus infections by each age group.

The frequency of multiple infections (31,4%) in all ages was significantly higher than single infection (45,2%) (*Z* = 5,069, *P* < 0,0001, 95% CI 0,2647–0,3636). This number is higher than the previous study in China (10,8%). [3] The viral coinfection in children under five years old in this study is slightly higher than the older one (52.3%). This result is the same as several other studies [18, 20, 22]. From the coinfection detected, the most frequent combination of viruses was ADV and HRV. The frequency and combination of multiple viruses detected are presented in Table 2.

Indonesia has five major islands with Java Island as the main island where 5 of 13 sentinels located. The total number of influenza-like illness cases in the first quarter of the year was greater than the rest of the year, as shown in Table 3. Respiratory viruses were more frequently detected in the early months of the year in all regions in Indonesia. However, the virus detection rate in each month was not significantly different. The increased respiratory infections during this period may be related to the rainy season in Indonesia which usually starts from October to April, annually. The humidity and temperature are assumed to be major factors, as described before [19, 31]. Respiratory viruses use aerosol as a mode of transmission. In the high humidity atmosphere as in tropical countries like Indonesia, the aerosol that contains respiratory viruses will survive longer.

Our recent study has several limitations. First, fewer patients <1 year old were enrolled. Specimens from young children tend to be hard to collect. There may have been selection bias, as surveillance staff may have avoided collecting swabs from this age group. Second, we only sampled patients visiting public health centers. Our findings may be limited to patients at private clinics or those who do not seek healthcare. Third, our study did not analyse the relationship among viruses, coinfection, and clinical features (severity). Further studies are encouraged to determine the type of viruses and coinfections that contribute to disease severity.

## 4. Conclusion

This study demonstrates a variety of respiratory viruses in all ages of patients which come to ILI sentinel. High incidence of multiple infections in ILI cases in Indonesia was detected from January to April 2012. The ILI surveillance system has provided valuable data of the other respiratory viral pathogen and can be used to provide more information to the clinicians. More detailed information is useful in future studies to provide a complete picture of respiratory viruses disease burden.

## Figures and Tables

**Table 1:** Viral etiology of ILI cases.

Virus	Total		Single		Multiple	
	*N*	(%)	*N*	(%)	*N*	(%)
Influenza						
Influenza A	63	18.75	27	42.86	36	57.14
Influenza B	58	17.26	25	43.10	33	56.90
Human rhinovirus	76	22.62	29	38.16	47	61.84
Human adenovirus	67	19.94	12	17.91	55	82.09
Respiratory syncytial virus						
RSV A	53	15.77	19	35.85	34	64.15
RSV B	4	1.19	1	25	3	75
Human enterovirus	36	10.71	13	36.11	23	63.89
Parainfluenza virus						
PIV 1	12	3.57	2	16.67	10	83.33
PIV 2	2	0.60	0	0	2	100
PIV 3	5	1.49	4	80	1	20
PIV 4	1	0.30	0	0	1	100
Human bocavirus	15	4.46	2	13.33	13	86.67
Human coronavirus						
Type NL63	6	1.79	2	33.33	4	66.67
Type OC43	4	1.19	1	25	3	75
Type 229 E	3	0.89	1	33.33	2	66.67

**Table 2:** Contribution of respiratory viruses as single or coinfections.

	ADV	Flu A	Flu B	HRV	RSV A	HEV
ADV	**(12)**					
Flu A	11	**(27)**				
Flu B	14	4	**(25)**			
HRV	20	17	11	**(29)**		
RSV A	13	13	10	13	**(19)**	
HEV	1	8	4	6	5	**(13)**

*Note*. Numbers in brackets indicate single infection.

**Table 3:** Geographic distribution of positive viral infections from influenza-like illness patients, by month of specimen collection.

Island	Jan	Feb	Mar	Apr	May	Jun	Jul	Aug	Sept	Total
Sumatera	12 (14)	3 (6)	8 (8)	9 (10)	0 (2)	1 (2)	2 (2)	1 (1)	0	**45**
Java	26 (33)	27 (35)	24 (30)	13 (18)	15 (18)	11 (13)	8 (8)	7 (7)	3 (4)	**166**
Kalimantan	5 (6)	17 (17)	8 (9)	13 (14)	10 (15)	4 (7)	7 (9)	0 (1)	3 (6)	**84**
Sulawesi	3 (3)	1 (1)	0 (1)	1 (3)	2 (3)	2 (3)	4 (4)	0 (2)	1 (1)	**21**
East Indo	4 (4)	2 (2)	1 (3)	1 (1)	0	1 (2)	0 (2)	1 (2)	2 (2)	**18**
*Total*										***334***

*Note*. Numbers in brackets indicate ILI patients.
